# Risk factors for hypertension and diabetes comorbidity in a Korean population: A cross-sectional study

**DOI:** 10.1371/journal.pone.0262757

**Published:** 2022-01-19

**Authors:** Jeong Hee Chi, Bum Ju Lee

**Affiliations:** 1 Department of Computer Science and Engineering, Konkuk University, Seoul, Republic of Korea; 2 Digital Health Research Division, Korea Institute of Oriental Medicine, Deajeon, Republic of Korea; Ehime University Graduate School of Medicine, JAPAN

## Abstract

**Background:**

Hypertension and diabetes are risk factors for severe cardiovascular disease and are prevalent comorbidities. No studies have examined the associations of various risk factors related to anthropometry, bone mineral density and body composition of specific body regions with hypertension and diabetes comorbidity (HDC). This study explored the association between HDC and various risk factors related to specific body regions.

**Method:**

A total of 7,978 subjects (3,451 men and 4,527 women) aged ≥ 50 years were included in the analysis. A binary logistic regression analysis based on complex sample data analysis was conducted to examine associations between the normal and diabetes groups, between the normal and hypertension groups, and between the normal and HDC groups.

**Results:**

Among Korean adults aged ≥ 50 years, 11.27% of men and 10.05% of women had both diabetes and hypertension. The HDC prevalence was higher in men than in women. In men, waist-to-height ratio (WHtR, adjusted OR = 1.63 [1.22–2.18], adjusted p<0.001) exhibited a more significant association with HDC than other indices, left leg fat mass (adjusted OR = 0.61 [0.45–0.81], adjusted p = 0.0009) and right leg fat mass (adjusted OR = 0.60 [0.45–0.81], adjusted p = 0.0007) displayed strong negative associations with diabetes, and left leg lean mass (adjusted OR = 0.77 [0.67–0.89], adjusted p = 0.0002) and trunk fat mass (adjusted OR = 1.28 [1.07–1.52], adjusted p = 0.0062) were significantly associated with hypertension. In women, left leg fat mass (adjusted OR = 0.29 [0.22–0.39], adjusted p<0.0001) and right leg fat mass (adjusted OR = 0.32 [0.23–0.45], adjusted p<0.0001) exhibited strong negative associations with HDC, waist circumference (WC) (adjusted OR = 2.15 [1.40–3.30], adjusted p = 0.0005) showed a stronger association with diabetes than did other indices, and WC (adjusted OR = 1.39 [1.11–1.74], adjusted p = 0.0040) and WHtR (adjusted OR = 1.39 [1.09–1.76], adjusted p = 0.0075) were significantly associated with hypertension.

**Discussion:**

HDC was more strongly associated with fat and lean mass than diabetes and hypertension. The association between HDC and body fat variables was more robust in women than in men.

## Introduction

Hypertension and diabetes are risk factors for the development of severe cardiovascular disease and are prevalent comorbidities [1–11]. The causal relationship between hypertension and diabetes is not clear. Several studies have reported that the prevalence of hypertension is high in diabetic patients [2, 12–18], and other studies have mentioned that the prevalence of diabetes is high in hypertensive patients [13, 14, 19, 20]. According to a study by Wang et al. [11] that included 14,422 participants aged 18–98 years in Changsha City, Hunan Province, 22.7% had hypertension, 7.0% had diabetes, and 3.8% had hypertension and diabetes comorbidities (HDC). Additionally, the researchers reported that among subjects over 50 years of age, the prevalence of hypertension was 33.6%, the prevalence of diabetes was 9.6%, and the prevalence of HDC was 5.8%. According to the Korea Hypertension Fact Sheet 2020, 29% of adults over 20 years have hypertension, and 36% of people with hypertension have diabetes [21]. The 2020 diabetes fact sheet in Korea reported that 13.8% of adults over 30 years had diabetes, and 61.3% with diabetes had hypertension [22]. Moreover, the report showed that among elderly individuals over 65 years, 27.6% had diabetes, and 74.3% of people with diabetes also had hypertension. As prior studies have shown, the prevalence of HDC increases significantly from middle age [11, 23, 24], and it is essential to carefully identify risk factors for HDC, which is associated with high rates of cardiovascular disease and mortality [1, 25].

Obesity is a risk factor for hypertension and diabetes. Indicators of obesity include body mass index (BMI), waist circumference (WC), and waist-to-height ratio (WHtR). BMI is a simple and objective measure of obesity that is calculated by dividing weight by the square of the height. Several studies have shown that BMI is a risk factor for diabetes [26, 27] and hypertension [27–30]. In addition, WC is an indicator that can reflect abdominal obesity well and has been mentioned as a risk factor for diabetes [31–34] and hypertension [35, 36]. However, WC does not consider height, and WHtR, which reflects the ratio of WC to height, has recently been reported as a risk factor for diabetes [37–43] and hypertension [30, 36, 44, 45]. These obesity-related indicators are also known as risk factors for HDC [46–48]. However, these obesity-related indicators cannot distinguish between body fat mass and muscle mass. Some studies have shown that the total body fat percentage or trunk fat and left and right leg fat are more strongly correlated with diabetes [49–52] or hypertension [49, 53–57] than obesity-related indicators. These previous studies showed that having a large amount of fat in the body is not unhealthy but rather that fat has different effects on health depending on which part of the body it is located. Several studies reported the importance of the total body fat mass or percentage indicating the amount of fat mass in the human body due to the association between total fat mass and cardiovascular diseases [49, 50, 52, 57–60]. Therefore, total body fat mass was considered to be an important risk factor for cardiovascular diseases. Furthermore, recent studies have suggested that cardiovascular diseases and mortality were more affected by fat mass distribution than fat mass itself [58–60]. Therefore, the position of fat distribution in the body was considered to be a risk factor for the diseases.

Although numerous studies have reported on anthropometric indices as risk factors for hypertension and diabetes, very few studies have analyzed the differences in anthropometric risk factors for HDC and for hypertension and diabetes separately. Furthermore, in the case of HDC, no studies have analyzed the relationship between HDC and various risk factors, such as anthropometric indices, bone mineral density (BMD), fat mass, and lean mass, in a specific region of body. This study aims to identify the association between HDC and various risk factors in specific body regions, such as anthropometrics, BMD, fat mass, and lean mass. Our findings are expected to be used as fundamental information for health management for patients with hypertension and diabetes or with both hypertension and diabetes.

## Material and methods

### Study population and data sources

The Korea National Health and Nutrition Examination Survey (KNHANES) is a nationally representative cross-sectional survey that is conducted by the Korean Ministry of Health and Welfare [61, 62] and has been carried out every year since 2007 to understand the health level of the Korean people. We used data from 2008–2011 (KNHANES IV-2, 3 and V-1, 2) in this study. From 2008 to 2011, KHANES included bone density and body fat tests performed on adults aged 20 years and older. Bone density and body fat tests were performed only from 2008 to 2011 throughout the entire screening survey period since 2007. The KNHANES datasets were approved by the Korea Ministry of Health and Welfare (2008-04EXP-01-C, 2009-01CON-03-2C, 2010-02CON-21-C, 2011-02CON-06-C) and conducted in accordance with the Declaration of Helsinki. In addition, we obtained ethics approval from the Institutional Review Board of Konkuk University (7001355-201802-E-063) and the Institutional Review Board of the Korea Institute of Oriental Medicine (I-2109/008-001) for the use of the KNHANES data. All subjects in this survey signed an informed consent form.

The KNHANES IV-2, 3 and V-1, 2 included 37,753 subjects who participated in a health interview survey and health examination. Information related to medical conditions, education, occupation, smoking, alcohol use and menopause was collected via face-to-face interviews and self-administered forms in the health interview survey. Variables such as height, weight, bone mineral density, and body fat and lean mass data were measured in the health examination. In this study, we targeted patients aged ≥ 50 years who underwent diabetes- and hypertension-related examinations.

### Definitions

Diabetes and hypertension were determined according to guidelines from the Korea Centers for Disease Control and Prevention [61, 62]. Diabetes was based on fasting plasma glucose≥126 mg/dL, self-reported current use of antidiabetic medication, or doctor-diagnosed diabetes. Hypertension was defined as systolic blood pressure (SBP)≥140 mmHg, diastolic blood pressure (DBP)≥90 mmHg, or self-reported current use of antihypertensive medication. HDC was defined as having both diabetes and hypertension.

### Measurements

Anthropometric measures such as height, weight, and WC were examined according to standard protocols. BMI was calculated as weight (kg)/square of height (m^2^). WHtR was calculated as WC divided by height. Fasting plasma glucose was assessed using a blood sample collected from the antecubital vein after an 8-hour fast. The BMD and body fat and lean composition were measured using dual-energy X-ray absorptiometry (DXA, DISCOVERY QDR-4500W fan-beam densitometer, Hologic, Inc., Bedford, MA, USA). BMD was measured in the left arm, right arm, left rib, right rib, thoracic spin, lumbar spine, pelvis, left leg and right leg. Body fat and lean mass were measured in the left arm, right arm, trunk, left leg, right leg, and whole body.

### Statistical analysis

All statistical analyses were performed after applying weights for health questionnaires and health examinations, as suggested by KNHANES, to retain a representative Korean population. Statistical analyses were performed using SPSS 21 for Windows (SPSS, Inc., Chicago, IL, USA). In this study, adults aged ≥ 50 years were divided into four groups: the normal group without either diabetes or hypertension, the diabetes group with diabetes and no hypertension, the hypertension group with hypertension and no diabetes, and the HDC group with both diabetes and hypertension. In crude analyses and in the analyses adjusted for covariates such as age, residential areas, education, occupation, alcohol consumption, smoking amount, menopause, and BMI, binary logistic regression analyses based on complex sample data analysis were conducted to identify associations between the normal group and the diabetes group, between the normal group and hypertension group, and between the normal group and HDC group. To evaluate sex differences in characteristics, we performed Rao-Scott chi-squared tests for categorical variables and t-tests for continuous variables using a general linear model. The significance level of α = 0.05 was applied for all statistical analyses, and odds ratios (95% confidence intervals) and p values are presented to indicate statistical significance. Table 1 shows the demographic characteristics and all variables analyzed in this study.

**Table 1:** Demographic characteristics.

Variables	Men				Women			
	Normal	HDC	Diabetes	Hypertension	Normal	HDC	Diabetes	Hypertension
Number of subjects	1,623	389	252	1,187	2,223	455	217	1,632
Age (mean, SE) ***	58.37 (0.25)	61.22 (0.56)	59.89 (0.60)	61.10 (0.33)	58.10 (0.23)	66.26 (0.49)	61.64 (0.61)	63.82 (0.29)
Residential area (ES (%), SE)								
City	70.19 (2.41)	74.45 (3.11)	73.97 (4.19)	72.40 (2.50)	73.73 (2.18)	71.22 (3.25)	76.79 (3.58)	69.07 (2.47)
Rural	29.81 (2.41)	25.55 (3.11)	26.03 (4.19)	27.60 (2.50)	26.27 (2.18)	28.78 (3.25)	23.21 (3.58)	30.93 (2.47)
Education (ES (%), SE) ***								
< = Elementary school	27.60 (1.40)	30.78 (2.96)	29.30 (3.60)	32.36 (1.83)	46.57 (1.42)	77.72 (2.44)	71.14 (3.67)	67.11 (1.57)
Middle school	22.90 (1.20)	24.06 (2.74)	21.60 (3.30)	19.35 (1.38)	20.77 (1.10)	8.42 (1.51)	14.49 (2.89)	14.11 (1.05)
High school	27.60 (1.50)	31.52 (3.19)	33.20 (3.50)	29.46 (1.75)	24.49 (1.19)	11.58 (1.89)	12.47 (2.79)	15.39 (1.18)
> = University	21.90 (1.50)	13.64 (2.32)	15.90 (2.80)	18.83 (1.53)	8.17 (0.86)	2.29 (0.83)	1.90 (0.77)	3.39 (0.55)
Occupation (ES (%), SE) ***								
White-collar worker	11.90 (1.10)	9.50 (2.20)	10.74 (2.17)	10.22 (1.20)	2.88 (0.43)	1.00 (0.53)	0.37 (0.37)	1.26 (0.29)
Office worker	5.80 (0.70)	3.80 (1.30)	2.91 (1.27)	4.18 (0.73)	2.18 (0.41)	0.65 (0.40)	1.12 (0.80)	0.66 (0.29)
Service	12.20 (1.20)	10.10 (2.10)	8.68 (2.09)	8.51 (1.03)	17.20 (1.14)	6.09 (1.42)	9.20 (2.19)	12.44 (1.12)
Farmer and fisher	18.30 (1.60)	12.20 (2.10)	13.78 (3.32)	15.12 (1.69)	11.44 (1.18)	10.97 (2.05)	12.18 (2.61)	10.57 (1.32)
Blue-collar worker	21.00 (1.50)	18.60 (2.60)	19.79 (3.35)	22.86 (1.98)	3.39 (0.47)	0.89 (0.60)	2.31 (1.16)	1.71 (0.34)
Elementary occupations	10.00 (0.90)	8.60 (1.80)	11.59 (2.61)	9.10 (0.98)	15.70 (1.03)	11.46 (1.77)	10.04 (2.75)	11.70 (0.96)
Unemployed (Housewife, etc.)	20.80 (1.30)	37.40 (3.00)	32.51 (3.55)	30.00 (1.69)	47.21 (1.41)	68.94 (2.94)	64.78 (3.80)	61.66 (1.70)
Alcohol consumption (ES (%), SE) ***								
Never drinker for a lifetime	7.55 (0.73)	6.21 (1.28)	7.04 (1.72)	7.37 (0.99)	27.82 (1.12)	39.78 (2.78)	32.98 (3.91)	34.76 (1.41)
Never drinker for 1 year	14.18 (1.07)	16.78 (2.19)	18.09 (2.67)	11.51 (1.09)	16.23 (0.95)	20.08 (2.11)	18.89 (3.19)	19.01 (1.20)
<1 per month	9.19 (0.94)	7.74 (1.78)	9.31 (1.85)	6.59 (0.93)	24.18 (1.11)	18.85 (2.23)	26.58 (3.55)	20.42 (1.31)
1 per month	7.00 (0.75)	7.65 (1.58)	7.55 (2.00)	6.84 (0.88)	10.35 (0.82)	7.46 (1.53)	13.35 (2.66)	8.73 (0.85)
2~4 per month	21.80 (1.32)	19.78 (2.76)	22.44 (3.34)	20.6 (1.58)	14.03 (0.95)	8.18 (1.60)	7.18 (2.22)	9.89 (0.92)
2~3 per week	22.77 (1.32)	14.47 (2.11)	17.41 (2.73)	24.26 (1.59)	5.38 (0.61)	3.27 (1.05)	0.69 (0.45)	4.33 (0.64)
> = 4 per week	17.52 (1.12)	27.37 (2.89)	18.16 (3.32)	22.84 (1.57)	2.00 (0.37)	2.38 (0.98)	0.33 (0.34)	2.85 (0.46)
Smoking amount (mean, SE) ***	7.04 (0.32)	6.32 (0.73)	8.02 (0.86)	5.55 (0.35)	0.52 (0.08)	0.61 (0.16)	0.46 (0.21)	0.45 (0.08)
Menopause (ES (%), SE)								
Yes	-	-	-	-	20.38 (1.46)	9.40 (2.25)	15.60 (3.80)	10.73 (1.41)
No	-	-	-	-	79.62 (1.46)	90.60 (2.25)	84.40 (3.80)	89.27 (1.41)
Anthropometrics (mean, SE)								
Height (cm) ***	167.8 (0.19)	166.7 (0.33)	167.0 (0.42)	167.0 (0.21)	155.0 (0.17)	152.9 (0.30)	153.9 (0.42)	153.0 (0.18)
Weight (kg) ***	65.60 (0.28)	70.50 (0.64)	66.91 (0.73)	68.19 (0.36)	56.45 (0.20)	60.28 (0.48)	58.32 (0.68)	58.07 (0.28)
Body mass index (kg/m^2^) *	23.26 (0.08)	25.34 (0.21)	23.92 (0.22)	24.41 (0.11)	23.49 (0.07)	25.74 (0.17)	24.59 (0.23)	24.75 (0.10)
Waist circumference (cm) ***	83.68 (0.24)	89.89 (0.52)	85.99 (0.56)	86.51 (0.30)	79.51 (0.23)	87.88 (0.52)	84.62 (0.67)	83.86 (0.29)
Waist-to-height ratio***	0.50 (0.00)	0.54 (0.00)	0.51 (0.00)	0.52 (0.00)	0.51 (0.00)	0.58 (0.00)	0.55 (0.00)	0.55 (0.00)
Bone mineral density (mean, SE)								
Left arm BMD (g/cm^2^) ***	0.81 (0.00)	0.82 (0.00)	0.81 (0.00)	0.82 (0.00)	0.65 (0.00)	0.64 (0.00)	0.64 (0.00)	0.64 (0.00)
Right arm BMD (g/cm^2^) ***	0.83 (0.00)	0.84 (0.00)	0.83 (0.00)	0.83 (0.00)	0.66 (0.00)	0.64 (0.00)	0.64 (0.00)	0.64 (0.00)
Left rib BMD (g/cm^2^) ***	0.69 (0.00)	0.72 (0.01)	0.70 (0.01)	0.70 (0.00)	0.61 (0.00)	0.60 (0.00)	0.59 (0.00)	0.59 (0.00)
Right rib BMD (g/cm^2^) ***	0.69 (0.00)	0.71 (0.01)	0.70 (0.01)	0.70 (0.00)	0.61 (0.00)	0.60 (0.00)	0.60 (0.01)	0.60 (0.00)
Thoracic spine BMD (g/cm^2^***)	0.93 (0.01)	0.98 (0.01)	0.96 (0.01)	0.95 (0.01)	0.78 (0.00)	0.77 (0.01)	0.78 (0.01)	0.76 (0.01)
Lumbar spine BMD (g/cm^2^) ***	1.05 (0.01)	1.10 (0.01)	1.07 (0.01)	1.08 (0.01)	0.97 (0.01)	0.94 (0.01)	0.94 (0.02)	0.95 (0.01)
Pelvis BMD (g/cm^2^) ***	1.08 (0.00)	1.14 (0.01)	1.11 (0.01)	1.11 (0.01)	1.01 (0.00)	0.97 (0.01)	0.99 (0.01)	0.97 (0.01)
Left leg BMD (g/cm^2^) ***	1.23 (0.01)	1.26 (0.01)	1.24 (0.01)	1.23 (0.01)	1.03 (0.00)	1.03 (0.01)	1.05 (0.02)	1.02 (0.01)
Right leg BMD (g/cm^2^) ***	1.23 (0.01)	1.25 (0.01)	1.24 (0.01)	1.24 (0.01)	1.04 (0.00)	1.04 (0.01)	1.05 (0.01)	1.02 (0.01)
Body total BMD (g/cm^2^) ***	1.16 (0.00)	1.19 (0.01)	1.17 (0.01)	1.17 (0.00)	1.04 (0.00)	1.01 (0.01)	1.02 (0.01)	1.01 (0.00)
Fat mass (mean, SE)								
Left arm fat (g) ***	720.8 (7.94)	852.5 (17.20)	742.6 (18.54)	806.6 (11.16)	1,149 (10.03)	1,283 (22.74)	1,232 (22.79)	1,218 (12.43)
Right arm fat (g) ***	728.0 (8.05)	884.6 (17.72)	777.2 (18.91)	821.8 (11.32)	1,155 (9.57)	1,312 (23.68)	1,244 (23.51)	1,227 (12.68)
Trunk fat (g) ***	7,744 (90.95)	10,078 (215.0)	8,440 (220.3)	9,058 (117.9)	9,873 (89.11)	12,303 (196.5)	11,156 (228.0)	11,163 (115.1)
Left leg fat (g) ***	1,886 (19.88)	2,061 (48.21)	1,811 (49.37)	2,044 (27.39)	2,948 (23.78)	2,697 (42.29)	2,719 (66.42)	2,962 (27.38)
Right leg fat (g) ***	1,926 (20.21)	2,133 (49.22)	1,853 (48.28)	2,091 (27.83)	3,021 (24.08)	2,788 (44.81)	2,785 (68.48)	3,028 (28.41)
Body total fat (g) ***	13,989 (142.0)	17,031 (337.1)	14,618 (342.4)	15,829 (191.0)	19,001 (144.7)	21,269 (300.0)	19,997 (367.5)	20,465 (179.4)
Lean mass (mean, SE)								
Left arm lean (g) ***	2,846 (14.06)	2,843 (28.94)	2,822 (39.86)	2,865 (18.13)	1,695 (8.17)	1,757 (15.41)	1,732 (22.42)	1,720 (8.22)
Right arm lean (g) ***	2,987 (14.86)	2,994 (31.02)	2,952 (45.69)	3,005 (18.11)	1,817 (8.19)	1,883 (17.77)	1,829 (23.07)	1,830 (8.62)
Trunk lean (g) ***	24,313 (106.6)	25,844 (223.2)	25,066 (264.0)	24,949 (119.6)	18.344 (67.77)	19,618 (144.3)	19,093 (196.7)	18,553 (77.05)
Left leg lean (g) ***	8,208 (39.91)	8,286 (83.00)	8,166 (94.43)	8,216 (45.99)	5,676 (23.56)	5,727 (48.06)	5,730 (68.35)	5,636 (26.90)
Right leg lean (g) ***	8,366 (41.48)	8,456 (74.46)	8,314 (101.4)	8,400 (47.20)	5,774 (24.21)	5,832 (50.63)	5,839 (69.11)	5,733 (27.71)
Body total lean (g) ***	50,992 (211.6)	52,802 (420.3)	51,616 (544.2)	51,743 (242.1)	37,008 (124.4)	38,540 (266.4)	37,898 (378.0)	37,151 (141.0)

**Notes**: The data are presented as the estimates of the population size (ES) ratio and standard error (SE) or as the mean and SE for categorical and continuous variables, respectively.

* p < 0.05, ** p < 0.001, *** p < 0.0001 indicate significant differences by Rao-Scott chi-squared tests for categorical variables and by t-tests for continuous variables using a general linear model between the men and women across the normal, HDC, diabetes, and hypertension groups.

**Abbreviations**: HDC, hypertension and diabetes comorbidity; BMD, bone mineral density; HDL, high-density lipoprotein.

## Results

### Participant characteristics

**Fig 1:**
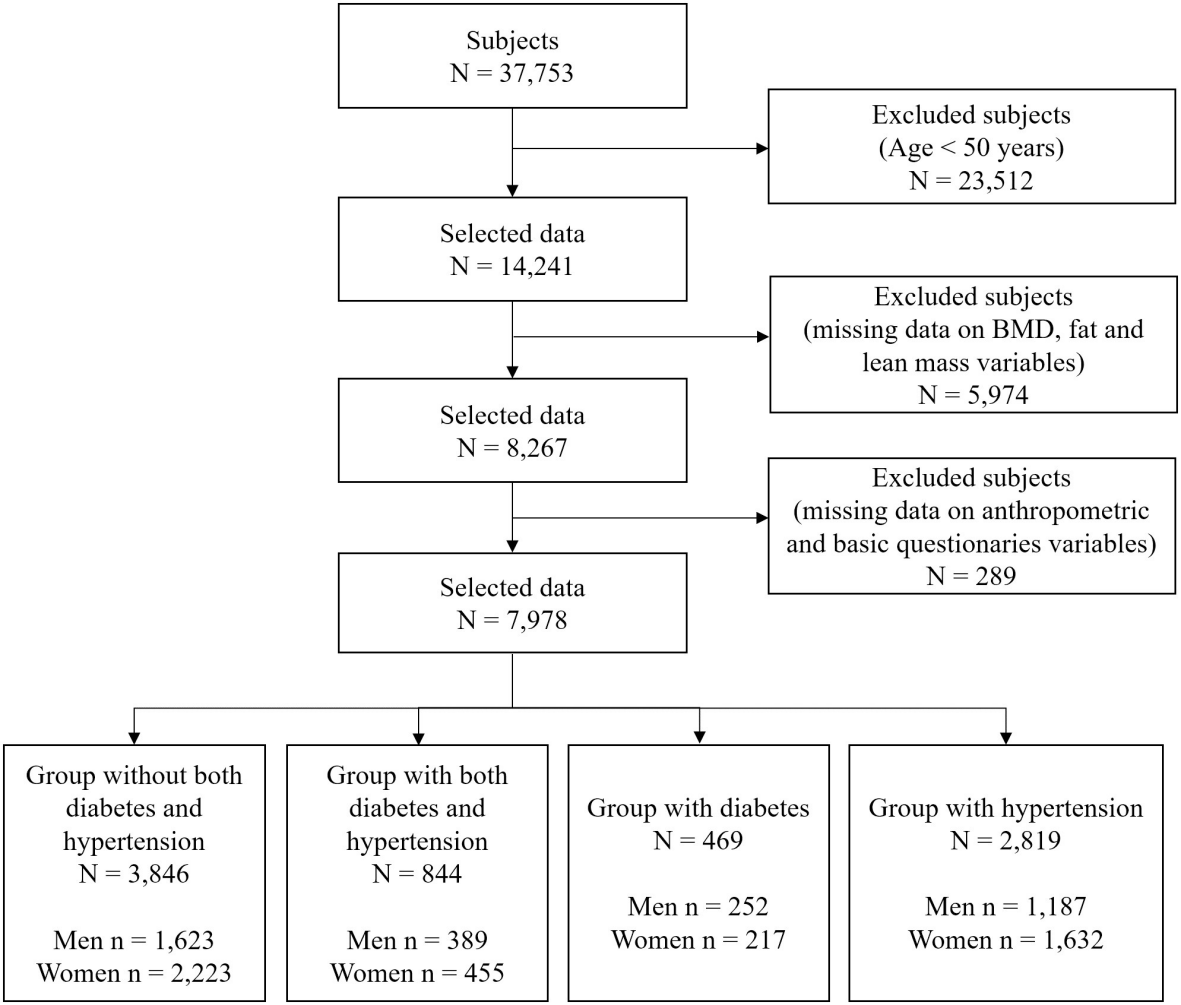
Sample selection procedure. BMD, bone mineral density.

After filtering according to various exclusion criteria, we selected 7,978 subjects. The detailed sample selection procedure is shown in Fig 1. The final sample consisted of 3,451 men (normal: 1,623, HDC: 389, diabetes: 252, hypertension: 1,187) and 4,527 women (normal: 2,223, HDC: 455, diabetes: 217, hypertension: 1,632).

Table 1 shows a description of the demographic characteristics and values for all variables. Among Korean adults aged ≥ 50 years, 11.27% of men and 10.05% of women had both diabetes and hypertension. The prevalence of HDC was higher in men than in women. In addition, the prevalence of diabetes and hypertension in men was 7.30% and 34.40%, respectively and that in women was 4.79% and 36.05%, respectively. The prevalence of diabetes was also higher in men than in women, and the prevalence of hypertension was higher in women than in men. Women were older than men in the HDC, diabetes and hypertension groups, and there was no significant difference between men and women in the normal group. In both men and women, BMI, WC, and WHtR were higher in the HDC group than in the diabetes and hypertension groups. In men, BMD was slightly higher in the HDC group than in the other groups, had similar values between the groups in women and was lower in women than in men. For both body fat and lean body mass in both men and women, the HDC group had higher values than the other groups, except for body fat mass in the left and right legs of women. For women, the left and right leg body fat mass in the HDC group was similar to that in the diabetes group but lower than that in the hypertension group. In particular, the difference in left and right body fat mass in the HDC group was more significant than that in the other groups.

### Associations of HDC, diabetes, and hypertension with anthropometric parameters, BMD, body fat and lean mass

Tables 2 and 3 show the associations between HDC, diabetes, and hypertension and anthropometric indices, BMD, body fat and lean mass in Korean men and women. As shown in the tables, the variables that were strongly associated with HDC, diabetes and hypertension were different. The odds ratios of variables associated with HDC were higher than those of variables associated with diabetes and hypertension. In addition, variables with strong associations were different in men and women.

**Table 2:** Associations of HDC, diabetes, and hypertension with anthropometrics, BMD, fat and lean mass in men.

Variables	Crude OR			Adjusted OR		
	HDC	Diabetes	Hypertension	HDC	Diabetes	Hypertension
Age	1.41 (1.22–1.61) ***	1.21 (1.04–1.4) *	1.38 (1.26–1.52) ***			
Anthropometrics						
Height	0.83 (0.73–0.95) *	0.88 (0.76–1.02)	0.87 (0.80–0.96) *	0.92 (0.78–1.08)	0.91 (0.77–1.08)	0.96 (0.87–1.07)
Weight	1.67 (1.44–1.94) ***	1.15 (0.98–1.35)	1.32 (1.20–1.45) ***	0.84 (0.61–1.16)	0.85 (0.59–1.21)	0.93 (0.75–1.16)
Body mass index	2.10 (1.79–2.47) ***	1.28 (1.08–1.52) *	1.53 (1.39–1.69) ***			
Waist circumference	2.30 (1.93–2.73) ***	1.36 (1.15–1.60) **	1.45 (1.32–1.60) ***	1.43 (1.09–1.88) *	1.27 (0.89–1.82)	0.87 (0.71–1.05)
Waist-to-height ratio	2.51 (2.12–2.98) ***	1.43 (1.22–1.69) ***	1.54 (1.40–1.69) ***	1.63 (1.22–2.18) **	1.44 (1.02–2.02) *	0.89 (0.74–1.08)
Bone mineral density						
Left arm BMD	1.06 (0.94–1.19)	1.00 (0.88–1.15)	1.03 (0.94–1.13)	0.94 (0.81–1.10)	0.98 (0.84–1.15)	0.98 (0.89–1.08)
Right arm BMD	1.19 (1.03–1.37) *	1.05 (0.90–1.24)	1.07 (0.97–1.18)	1.07 (0.90–1.28)	1.04 (0.87–1.25)	1.04 (0.93–1.17)
Left rib BMD	1.30 (0.99–1.72)	1.13 (0.97–1.32)	1.10 (0.98–1.24)	1.11 (0.95–1.29)	1.10 (0.95–1.28)	0.99 (0.89–1.11)
Right rib BMD	1.37 (1.19–1.58) ***	1.12 (0.95–1.32)	1.14 (1.03–1.25) *	1.14 (0.97–1.35)	1.04 (0.88–1.24)	1.03 (0.92–1.14)
Thoracic spine BMD	1.42 (1.23–1.65) ***	1.21 (1.03–1.41) *	1.15 (1.05–1.27) *	1.18 (1.02–1.36) *	1.14 (0.98–1.34)	1.03 (0.93–1.14)
Lumbar spine BMD	1.25 (1.11–1.41) **	1.13 (0.98–1.29)	1.16 (1.05–1.28) *	1.07 (0.94–1.20)	1.06 (0.93–1.21)	1.07 (0.96–1.18)
Pelvis BMD	1.41 (1.23–1.63) ***	1.21 (1.04–1.42) *	1.21 (1.11–1.32) ***	1.26 (1.03–1.53) *	1.20 (1.02–1.42) *	1.13 (1.01–1.27) *
Left leg BMD	1.16 (1.03–1.31) *	1.07 (0.94–1.21)	1.02 (0.94–1.12)	1.00 (0.85–1.18)	1.00 (0.87–1.14)	0.94 (0.85–1.03)
Right leg BMD	1.13 (1.02–1.24) *	1.06 (0.95–1.18)	1.03 (0.94–1.12)	0.97 (0.88–1.08)	1.00 (0.89–1.13)	0.93 (0.85–1.02)
Body total BMD	1.21 (1.05–1.39) *	1.07 (0.91–1.25)	1.04 (0.95–1.14)	1.07 (0.91–1.25)	1.02 (0.86–1.19)	0.98 (0.88–1.08)
Fat mass						
Left arm fat	1.69 (1.47–1.95) ***	1.09 (0.94–1.28)	1.41 (1.28–1.54) ***	1.02 (0.84–1.24)	0.85 (0.66–1.09)	1.09 (0.95–1.25)
Right arm fat	1.85 (1.60–2.13) ***	1.22 (1.05–1.43) *	1.44 (1.31–1.59) ***	1.16 (0.94–1.43)	1.02 (0.78–1.33)	1.11 (0.96–1.30)
Trunk fat	2.12 (1.81–2.48) ***	1.26 (1.09–1.47) *	1.56 (1.42–1.72) ***	1.61 (1.24–2.08) **	1.12 (0.80–1.55)	1.28 (1.07–1.52) *
Left leg fat	1.31 (1.13–1.51) ***	0.88 (0.74–1.04)	1.28 (1.17–1.41) ***	0.68 (0.56–0.83) **	0.61 (0.45–0.81) **	0.95 (0.83–1.09)
Right leg fat	1.36 (1.18–1.57) ***	0.89 (0.75–1.05)	1.29 (1.17–1.42) ***	0.71 (0.59–0.86) **	0.60 (0.45–0.81) **	0.94 (0.82–1.08)
Body total fat	1.90 (1.63–2.21) ***	1.15 (0.99–1.34)	1.50 (1.36–1.65) ***	1.18 (0.92–1.52)	0.87 (0.62–1.22)	1.16 (0.98–1.38)
Lean mass						
Left arm lean	0.99 (0.86–1.15)	0.94 (0.78–1.14)	1.04 (0.95–1.15)	0.70 (0.57–0.85) **	0.83 (0.66–1.05)	0.93 (0.82–1.05)
Right arm lean	1.02 (0.88–1.17)	0.93 (0.76–1.13)	1.04 (0.94–1.14)	0.73 (0.61–0.88) *	0.80 (0.62–1.04)	0.95 (0.84–1.07)
Trunk lean	1.58 (1.36–1.83) ***	1.25 (1.06–1.48) *	1.22 (1.11–1.35) ***	1.10 (0.90–1.36)	1.23 (0.96–1.58)	1.02 (0.87–1.20)
Left leg lean	1.07 (0.92–1.24)	0.97 (0.82–1.14)	1.01 (0.92–1.10)	0.64 (0.53–0.78) ***	0.79 (0.64–0.97) *	0.77 (0.67–0.89) **
Right leg lean	1.08 (0.94–1.23)	0.96 (0.81–1.14)	1.03 (0.94–1.13)	0.65 (0.54–0.79) ***	0.78 (0.63–0.97) *	0.80 (0.69–0.92) *
Body total lean	1.32 (1.15–1.53) **	1.10 (0.92–1.31)	1.13 (1.02–1.24) *	0.80 (0.65–0.98) *	0.96 (0.74–1.24)	0.89 (0.76–1.04)

Notes:

* p < 0.05,

** p < 0.001,

*** p < 0.0001.

The results were obtained from the crude analysis and analyses adjusted for age, residential area, education, occupation, alcohol consumption, smoking amount, and BMI using complex sample binary logistic regression in SPSS, with normal group vs. HDC group, normal group vs. diabetes group, and normal group vs. hypertension group.

**Abbreviations**: HDC, hypertension and diabetes comorbidity; BMD, bone mineral density; OR, odds ratio.

**Table 3:** Associations of HDC, diabetes and hypertension with anthropometrics, BMD, fat and lean mass in women.

Variables	Crude OR			Adjusted OR		
	HDC	Diabetes	Hypertension	HDC	Diabetes	Hypertension
Age	2.42 (2.14–2.73) ***	1.52 (1.32–1.75) ***	1.90 (1.75–2.07)***			
Anthropometrics						
Height	0.70 (0.63–0.79) ***	0.83 (0.71–0.97) *	0.72 (0.66–0.78) ***	1.25 (1.01–1.54) *	1.10 (0.85–1.43)	1.02 (0.89–1.17)
Weight	1.62 (1.42–1.84) ***	1.29 (1.08–1.54) *	1.22 (1.12–1.33) ***	1.52 (1.03–2.25) *	1.21 (0.73–1.99)	1.06 (0.81–1.38)
Body mass index	2.12 (1.87–2.42) ***	1.49 (1.26–1.77) ***	1.55 (1.42–1.69) ***			
Waist circumference	2.75 (2.39–3.16) ***	1.93 (1.62–2.30) ***	1.72 (1.58–1.88) ***	2.35 (1.65–3.35) ***	2.15 (1.40–3.30) **	1.39 (1.11–1.74) *
Waist-to-height ratio	3.04 (2.64–3.50) ***	2.00 (1.69–2.36) ***	1.92 (1.76–2.10) ***	1.90 (1.33–2.73) **	1.88 (1.24–2.86) *	1.39 (1.09–1.76) *
Bone mineral density						
Left arm BMD	0.82 (0.72–0.92) *	0.82 (0.71–0.95) *	0.81 (0.74–0.88) ***	1.24 (1.05–1.47) *	1.03 (0.85–1.25)	1.11 (0.98–1.26)
Right arm BMD	0.76 (0.67–0.86) ***	0.77 (0.66–0.90) **	0.78 (0.71–0.85) ***	1.24 (1.01–1.52) *	1.00 (0.79–1.27)	1.15 (0.98–1.34)
Left rib BMD	0.90 (0.80–1.01)	0.83 (0.71–0.96) *	0.84 (0.77–0.91) ***	1.15 (0.95–1.39)	0.86 (0.67–1.09)	1.09 (0.94–1.27)
Right rib BMD	0.86 (0.77–0.96) *	0.90 (0.77–1.04)	0.82 (0.76–0.89) ***	1.17 (0.95–1.45)	1.01 (0.77–1.33)	1.10 (0.94–1.29)
Thoracic spine BMD	0.93 (0.82–1.04)	0.95 (0.81–1.10)	0.87 (0.80–0.95) *	1.51 (1.22–1.86) **	1.11 (0.86–1.44)	1.14 (0.99–1.31)
Lumbar spine BMD	0.87 (0.73–1.04)	0.84 (0.68–1.04)	0.91 (0.84–1.00) *	1.03 (0.89–1.20)	0.93 (0.74–1.16)	0.99 (0.88–1.11)
Pelvis BMD	0.78 (0.68–0.90) **	0.86 (0.73–1.01)	0.81 (0.75–0.87) ***	1.03 (0.88–1.20)	0.89 (0.73–1.08)	1.00 (0.87–1.15)
Left leg BMD	1.00 (0.85–1.17)	1.11 (0.93–1.32)	0.91 (0.82–0.99) *	1.17 (1.00–1.37)	1.30 (1.11–1.53) **	1.03 (0.91–1.16)
Right leg BMD	1.03 (0.88–1.21)	1.09 (0.91–1.30)	0.92 (0.83–1.01)	1.13 (0.97–1.32)	1.34 (1.11–1.60) *	1.08 (0.96–1.21)
Body total BMD	0.79 (0.68–0.90) **	0.83 (0.70–0.98) *	0.76 (0.70–0.82) ***	1.11 (0.90–1.36)	1.11 (0.87–1.42)	0.97 (0.84–1.11)
Fat mass						
Left arm fat	1.48 (1.30–1.69) ***	1.31 (1.12–1.53) **	1.23 (1.12–1.34) ***	0.99 (0.73–1.34)	0.91 (0.66–1.25)	0.81 (0.66–1.01)
Right arm fat	1.55 (1.36–1.77) ***	1.33 (1.14–1.54) **	1.23 (1.13–1.34) ***	1.20 (0.90–1.61)	0.99 (0.74–1.31)	0.90 (0.73–1.10)
Trunk fat	2.15 (1.87–2.47) ***	1.55 (1.32–1.83) ***	1.52 (1.39–1.67) ***	1.83 (1.27–2.64) *	1.71 (1.08–2.71) *	1.29 (1.01–1.64) *
Left leg fat	0.70 (0.61–0.81) ***	0.72 (0.59–0.89) *	1.02 (0.93–1.11)	0.29 (0.22–0.39) ***	0.50 (0.33–0.77) *	0.74 (0.62–0.88) **
Right leg fat	0.73 (0.64–0.84) ***	0.73 (0.59–0.89) *	1.01 (0.93–1.10)	0.32 (0.23–0.45) ***	0.50 (0.34–0.73) **	0.73 (0.61–0.88) **
Body total fat	1.58 (1.39–1.80) ***	1.24 (1.05–1.47) *	1.34 (1.23–1.47) ***	0.79 (0.54–1.16)	0.92 (0.60–1.41)	0.94 (0.72–1.22)
Lean mass						
Left arm lean	1.25 (1.11–1.39) **	1.14 (0.97–1.35)	1.10 (1.02–1.19) *	1.23 (1.01–1.49) *	1.01 (0.78–1.31)	1.04 (0.91–1.19)
Right arm lean	1.26 (1.11–1.43) **	1.05 (0.87–1.25)	1.05 (0.97–1.13)	1.31 (1.07–1.61) *	0.92 (0.71–1.19)	1.04 (0.92–1.18)
Trunk lean	1.70 (1.50–1.93) ***	1.38 (1.17–1.63) **	1.10 (1.01–1.19) *	1.99 (1.50–2.64) ***	1.46 (1.07–2.00) *	1.19 (1.00–1.40) *
Left leg lean	1.07 (0.94–1.21)	1.07 (0.90–1.27)	0.95 (0.88–1.03)	1.10 (0.89–1.36)	1.08 (0.82–1.41)	0.99 (0.86–1.14)
Right leg lean	1.07 (0.94–1.22)	1.08 (0.91–1.28)	0.95 (0.88–1.03)	1.12 (0.89–1.41)	1.11 (0.86–1.45)	1.02 (0.88–1.19)
Body total lean	1.41 (1.25–1.60) ***	1.23 (1.03–1.46) *	1.03 (0.95–1.12)	1.61 (1.24–2.10) **	1.25 (0.91–1.71)	1.11 (0.94–1.30)

Notes:

* p < 0.05,

** p < 0.001,

*** p < 0.0001.

The results were obtained from the crude analysis and analyses adjusted for age, residential areas, education, occupation, alcohol consumption, smoking amount, menopause and BMI using complex sample binary logistic regression on SPSS, with normal group vs. HDC group, normal group vs. Diabetes group, and normal group vs. Hypertension group.

**Abbreviations**: HDC, hypertension and diabetes comorbidity; BMD, bone mineral density; OR, odds ratio.

In men, in the crude analysis, WHtR exhibited a strong association with HDC (odds ratio (OR) = 2.51 [confidence interval = 2.12–2.98], p<0.0001) and diabetes (OR = 1.43 [1.22–1.69], p<0.0001) among all variables examined in this study. However, trunk fat mass (OR = 1.56 [1.42–1.72], p<0.0001) showed a stronger association with hypertension than did other indices. After adjusting for age, residence area, education, occupation, alcohol consumption, smoking amount, and BMI, WHtR still showed a strong association with HDC (adjusted OR = 1.63 [1.22–2.18], adjusted p<0.001). Left leg fat (adjusted OR = 0.61 [0.45–0.81], adjusted p = 0.0009) and right leg fat (adjusted OR = 0.60 [0.45–0.81], adjusted p = 0.0007) exhibited strong negative associations with diabetes, and trunk fat (adjusted OR = 1.28 [1.07–1.52], adjusted p = 0.0062) and left leg lean mass (adjusted OR = 0.77 [0.67–0.89], adjusted p = 0.0002) displayed a strong positive and negative association with hypertension, respectively.

In women, among all the variables, WHtR displayed a stronger association with all groups than did other indices in the crude analyses: HDC (OR = 3.04 [2.64–3.50], p<0.0001), diabetes (OR = 2.00 [1.69–2.36], p<0.0001), and hypertension (OR = 1.92 [1.76–2.10], p<0.0001). After adjusting for age, residential area, education, occupation, alcohol consumption, smoking amount, menopause and BMI, left leg fat and right leg fat showed strong negative associations with HDC (left leg fat: adjusted OR = 0.29 [0.22–0.39], adjusted p<0.0001, right leg fat: adjusted OR = 0.32 [0.23–0.45], adjusted p<0.0001), WC exhibited the most significant association with diabetes (adjusted OR = 2.15 [1.40–3.30], adjusted p = 0.0005), and WC and WHtR displayed strong associations with hypertension (WC: adjusted OR = 1.39 [1.11–1.74], adjusted p = 0.0040, WHtR: adjusted OR = 1.39 [1.09–1.76], adjusted p = 0.0075).

Based on the adjusted analysis, for men, HDC was more likely to be associated with anthropometrics, whereas diabetes was more likely to be associated with fat mass and hypertension with lean body mass. For women, HDC was more likely to be associated with fat mass, whereas diabetes and hypertension were more likely to be associated with anthropometrics.

## Discussion

Various anthropometric indices have different effects on health. This study aims to analyze the relationship between various anthropometric indices and HDC. Hypertension and diabetes are closely related to each other and often coexist [2, 12, 13, 15–20, 63, 64]. Several studies have noted that hypertension is the most common complication of diabetes [2, 12–18]. Notably, Landsberg and Molitch [65] described that hypertension occurs in 50% to 80% of patients with diabetes in the US. Cheung [66] noted that in the Hong Kong Cardiovascular Risk Factor Prevalence Study, 58% of people with diabetes had hypertension, and 44% of people with hypertension had diabetes. Petrie et al. [14] indicated that patients with diabetes are twice as likely to have hypertension as those without diabetes, and Sabuncu et al. [18] noted that in Turkey, 67.5% of adult patients with diabetes have hypertension. Other studies indicated that subjects with hypertension have a greater risk of developing diabetes than those with normal blood pressure [13, 14, 19, 20]. According to a prospective cohort study conducted in the United States, diabetes was almost 2.5 times as likely to develop in subjects with hypertension than in subjects with normal blood pressure [13]. In addition, some studies described that while diabetes may causally affect hypertension, hypertension is likely not the cause of diabetes [17]. As seen in previous studies, diabetes and hypertension are closely related, but the causal relationship is still controversial. According to the 2019 Korean Health Statistics, the prevalence of hypertension among adults over 30 years in Korea is 27.2%, and the prevalence of diabetes is 11.8% [67]. Since the prevalence of hypertension is higher than that of diabetes, we expected that the risk indicator in the HDC group would be the same as that in the hypertension group. However, our results differed from our expectations. The variable with the highest association with HDC was different from that for diabetes and hypertension in both men and women. There was also a difference in the associations of the variables between men and women.

Regarding HDC and anthropometric risk factors, Tarleton et al. [68] examined anthropometric risk factors for hypertension, diabetes, and HDC in several ethnic groups and reported that BMI and WC were positively associated with diabetes, hypertension and HDC in whites and African Americans. BMI was not a useful indicator of hypertension, diabetes, or HDC in Asians. Wang et al. [11] assessed the association of BMI and WC with diabetes, hypertension, and HDC. They argued that WC was a better indicator than BMI for HDC. Tiptaradol and Aekplakorn [69] tested the association between sociodemographic factors and the coexistence of hypertension and diabetes in the Thai population and suggested that risk factors related to the coexistence of hypertension and diabetes were age, low education level, living in urban areas, and WC. Additionally, they argued that BMI was not associated with the coexistence of hypertension and diabetes, whereas WC was associated with the coexistence of the diseases. A study by Shumye et al. [70] documented that risk factors for diabetes among hypertensive patients were WHtR and WC in Northeast Ethiopia. Additionally, according to an analysis of data from 882 subjects in Kuala Lumpur, Malaysia, the strongest risk factor for cardiovascular disease (CVD), a complication of diabetes and hypertension, was waist-related measurements in men and BMI, WHtR, and WC in women [46]. According to a cross-sectional study of diabetic adults 28–75 years old in the Diabetes Clinic of Golestan Hospital, Ahvaz, Khuzestan Province, Iran, there was a strong association between WC and hypertension prevalence in diabetic patients [47]. Saleem et al. [48] suggested that hypertension is closely related to obesity in individuals with diabetes and that hypertension is more likely to be present as weight and obesity increase in individuals with diabetes in Lahore, Pakistan. Our results are in agreement with the results of previous studies [11, 46, 47, 68–70] related to HDC, indicating that WHtR and WC were better indicators than other anthropometric indices in men and women and that BMI was an insufficient predictor of HDC. However, these indicators related to obesity are indicators that cannot distinguish between body fat and muscle mass, and other indicators that can better reflect body information are required.

With respect to risk indicators for diabetes or hypertension, several studies have noted that obesity-related indicators such as BMI, WC, and WHtR are strongly associated with diabetes and hypertension. First, concerning risk indicators associated with diabetes, Tulloch-Reid et al. [26] argued that BMI was a significant predictor of diabetes risk according to a 5.25-year follow-up study of Pima Indians. In addition, according to the results of a study by Chei et al. [27], at the 10-year follow-up for hypertension and diabetes, the excellent predictor for diabetes was BMI, and those for hypertension were BMI and WC. Freemantle et al. [32] argued that WC was a good indicator for predicting diabetes. Kapoor et al. [33] noted that WC could better stratify individuals according to diabetes status than BMI in an Indian population. Issaka et al. [34] argued that WC might be the indicator most helpful in identifying those with diabetes among West African adults. However, recently, several studies introduced WHtR as a risk indicator for diabetes because it is reasonable to assume that short subjects generally have more risk factors related to abdominal fat than tall subjects with a similar WC [37–43]. Bell et al. [28] noted that BMI was associated with a higher prevalence of hypertension in a cross-sectional study of adults aged 30–65 years in China, the Philippines, and the United States. Chei et al. [27] argued that the excellent predictors of hypertension were BMI and WC according to the 10-year follow-up study for hypertension and diabetes. Landi et al. [29] suggested that BMI could directly affect blood pressure regardless of other clinical risk factors according to the Longevity Check-up 7+ project in Italy. Hu et al. [30] argued that BMI was an excellent predictor among participants aged ≥65 years according to a cross-sectional survey conducted in Jiangxi Province, China, from 2013 to 2014. Gus et al. [35] noted that WC was a better indicator for hypertension than BMI in Porto Alegre Sangrós et al. [36] showed that hypertension had the strongest association with WHtR in women and WC in men, according to a cross-sectional analysis of 2022 participants in the PREDAPS study. In the case of participants aged < 65 years, Hu et al. [30] argued that WHtR was an excellent predictor of hypertension. Lu et al. [45] suggested that WHtR was positively associated with hypertension, particularly among subjects aged <60 years in southern China. According to our findings, both WC and WHtR were associated with all groups in women and with HDC in men and, only WHtR was associated with diabetes; no significant association was found for hypertension.

Regarding body composition indices, such as fat mass, lean mass and BMD in individuals with diabetes and hypertension, several studies have argued that it is more critical to maintain proper body fat than BMI to prevent diabetes [9, 50, 51]. Gómez-Ambrosi et al. [50] showed that body fat percentage was a more important determinant of diabetes than BMI and WC in a cross-sectional analysis of 4,828 white subjects. In an observational cohort study conducted with data drawn from the Shanghai Diabetes Study, Zhao et al. [51] noted that maintaining adequate body fat is more important than managing BMI for preventing diabetes. In our previous study of Korean adults over 40 years of age, diabetes was highly associated with WHtR in men and left leg and right leg fat in women [52]. In a study of Korean older adults, Han et al. [53] argued that hypertension was strongly associated with sarcopenia, especially in subjects with diabetes, and the relationship between hypertension and sarcopenia was strong. In an analysis of Scottish Health Surveys and Health Surveys for England, Han et al. [54] showed that body fat percentage had a much stronger association with hypertension than BMI. Han et al. [55] also suggested that body fat and skeletal muscle are strongly associated with diabetes and may be more useful indicators than BMI. Takase reported that the fat mass index calculated as the fat mass divided by the height squared was strongly associated with hypertension in a cross-sectional study of 5,058 men and 11,842 women aged ≥ 20 years in the Miyagi Prefecture, northeastern Japan [57]. With respect to BMD, Dennison et al. [71] argued that the total femur and femoral neck BMD exhibited a positive association with insulin resistance, with a stronger association in women. Strotmeyer et al. [72] reported that diabetes was correlated with higher hip BMD and lower spine bone volume in 2,970 white and black men and women aged 70–79 years. Ma et al. [73] found that patients with diabetes had high BMD of the femoral neck, hip, and spine. Our findings are consistent with the results of previous studies [50, 54, 55], indicating that body fat had a stronger association with diabetes and hypertension than WC in men. However, our finding contrasted with that of previous studies showing that in women, WC was more associated with diabetes and hypertension than body fat mass.

Additionally, diabetes and hypertension are highly associated with cardiometabolic disease. Lee et al. [74] reported that WHtR was more strongly associated with cardiometabolic disease than WC or BMI in both sexes based on an analysis of 10 published studies. Peppa et al. [75] showed that DXA indices of central and peripheral fat distribution were strongly associated with cardiometabolic disease in a sample of 150 postmenopausal women. Wu et al. [76] showed that the percentage of lower-body fat mass was correlated with cardiometabolic disease in both sexes in a sample of 207 Japanese adults. Our findings on diabetes and hypertension support the findings of previous studies that the WHtR and body fat mass are highly correlated with cardiometabolic disease, which has diabetes and hypertension-related factors as risk indicators. In addition, our results suggest that among elderly individuals in Korea, men and women may have different essential indicators of cardiometabolic disease. The strong association of HDC with WHtR and trunk fat in men suggests that abdominal-related indicators should be managed more closely, while the strong association of left leg fat and right leg fat with HDC in women suggests the need to manage leg-related indicators.

This study has several limitations. First, due to the cross-sectional design, it is difficult to determine causality. Second, we cannot guarantee that our findings reflect the situation in other countries or ethnic groups because of differences in socioeconomic and environmental characteristics. Despite these limitations, our study has powerful statistical results because KNHANES data reflect a nationally representative sample of the Korean population.

In conclusion, in the present study, we evaluated the associations of HDC, diabetes and hypertension with various body indicators, such as anthropometrics, BMD, body fat, and lean mass, in the Korean population. WHtR and trunk fat in men and left leg and right leg fat in women showed significant associations with HDC, left leg and right leg fat in men and WC in women exhibited stronger associations with diabetes than did other indices, and left leg lean mass in men and WC and WHtR in women showed significant associations with hypertension. In particular, HDC was more associated with body fat and lean mass variables than diabetes and hypertension. Our findings provide information that may be useful for the identification of HDC during initial screening steps. Further studies are needed to build a model for accurate identification based on a combination of anthropometrics, BMD, body fat mass, and lean body mass.
